# Implementation of healthcare accreditation in Danish general practice: a questionnaire study exploring general practitioners’ perspectives on external support

**DOI:** 10.1080/02813432.2021.1882084

**Published:** 2021-03-01

**Authors:** Maria Luisa Overgaard Jensen, Flemming Bro, Anna Mygind

**Affiliations:** a Department of Public Health, Aarhus University, Aarhus C, Denmark; b Research Unit for General Practice, Aarhus C, Denmark

**Keywords:** General practice, implementation science, primary health care, accreditation, quality improvement, support, Denmark

## Abstract

**Objective:**

To describe the use and perceived usefulness of implementation support provided to general practice during an accreditation process and to explore potential variations across clinic characteristics.

**Design:**

Cross-sectional questionnaire study.

**Setting and subjects:**

All Danish general practice clinics undergoing an accreditation survey from 27 September 2016 to 15 December 2017 (*n* = 608).

**Main outcome measures:**

Use and perceived usefulness of seven types of implementation support as reported by general practitioners (GPs). Clinic characteristics included practice type, number of GP partners and staff and employment of GP trainees.

**Results:**

The total response rate was 74% (*n* = 447). Most clinics (99.5%) used some type of implementation support (average: 4.8 different types). The most used types of support were peer support (80–92%) and various accreditation documents (85–92%). Support tailored to the individual clinic was most often considered useful (91–97%). However, this type of support was used relatively infrequently (16–40%). In most cases, clinic characteristics were neither significantly associated with the use of support nor with the perceived usefulness of the available support.

**Conclusion:**

During the accreditation processes, each clinic used a broad variety of implementation support. Support tailored to the individual clinic was highly appreciated and should be promoted in future quality interventions in general practice. Discussions with peers were widely used, and it should be investigated further how peer discussions are best facilitated. The study calls for a multifactorial approach to future quality interventions in general practice to target the needs and capacities of the individual clinics.

## Introduction

Accreditation can be defined as a procedure in which a recognised external institution evaluates an organisation based on a predefined set of quality standards [1]. Accreditation has long been used in the healthcare sector. One of the first examples was seen at a United States hospital in 1919 [1]. Since then, accreditation has been used extensively in the secondary healthcare sector [1–3]. It was not until the early 1990s that the first accreditation programme for general practice was introduced in Australia [2]. Thereafter, several countries introduced accreditation in general practice, including Germany, the Netherlands and the United Kingdom. In 2010, the Organisation of General Practitioners in Denmark and the Danish Regions decided that all Danish general practice clinics should undergo mandatory accreditation, and this was scheduled to occur from January 2016 to December 2018 [4].

The accreditation of general practice in Denmark was based on the Danish Healthcare Quality Programme, which consisted of 16 quality standards adapted to the context of general practice [5]. The quality standards covered professional, organisational and patient-perceived quality [5]. The core of accreditation was the survey [5]. The clinic was advised one year in advance about the date of this survey. On this date, the clinic received a visit from two surveyors: a general practitioner (GP) and a clinic staff. The surveyors prepared a report based on their assessment; this report was used by the accreditation board to decide whether the clinic was eligible for accreditation [5].

Unlike accreditation schemes in many other countries [2], the accreditation of general practice was mandatory in Denmark [5]. However, the individual clinic could decide how to implement the quality standards [5]. According to the collective agreement between the Danish Organisation of General Practitioners and the Danish Regions, the regions were to deliver implementation support to the clinics [6]. A qualitative study has shown that some clinics used the regional support to increase their understanding of the accreditation standards, but their experiences with the provided support were mixed. This study also found that some clinics supplemented the regional support with informal support from experts and colleagues [6]. We know from other quality interventions that practice facilitation and peer discussion can be useful [7,8], but it remains unknown whether these tools are used and perceived as useful in the accreditation process.

Other studies have shown that almost half of the Danish GPs had negative attitudes towards accreditation prior to its implementation [9] and that some GPs increased their intrinsic motivation during the accreditation process, perhaps due to the possibilities for displaying professional competence and/or improving the quality of their work [10]. The accreditation process in the clinic thus seemed to be successful, but some clinics found it hard to understand the accreditation standards [6].

Previous research has found that the implementation of accreditation programmes depends on the context, including how well the accreditation programme is received and whether the clinics are capable of embracing accreditation [11].

Hence, the aim of this study was to describe, from a GP perspective, the use and perceived usefulness of the implementation support provided to general practice during the accreditation process and to explore potential variations across clinic characteristics.

## Material and methods

### Setting

Danish healthcare is mainly tax financed, with free-of-charge access to general practice [12]. GPs are private entrepreneurs who are mostly financed through the public healthcare reimbursement scheme and services are regulated by collective agreements between the Danish Regions and the Organisation of General Practitioners in Denmark [12]. There is approximately 3500 GPs and almost 2000 clinics in Denmark [13]. The clinics are organised in different practice types, depending on whether the clinic is owned by one or several GPs and the degree of cooperation with other GPs. Almost every clinic has staff members and approximately half of the clinics have GP trainees [13].

### Study design and population

All Danish general practice clinics completing their accreditation survey between 27 September 2016 and 15 December 2017 received an article-based questionnaire on their experiences with the accreditation process. In total, 608 clinics completed their accreditation survey during the study period. The questionnaire was handed out by the visiting GP surveyor immediately after completion of the survey. The surveyor invited the GP who was most deeply involved in the accreditation to answer the questionnaire on behalf of the clinic.

### Data collection

The pilot test was conducted among 14 participants, including surveyors, GPs who had just completed their accreditation survey and experienced researchers in primary healthcare. Eight of these informants gave feedback after completing the questionnaire, whereas six of them gave verbal feedback to the interviewer (using cognitive interviewing techniques) during the completion of the questionnaire [14]. The questions regarding implementation support were developed based on a mapping from 2016 which identified the types of implementation support delivered by the regions in the first year of accreditation [15]. Other types of support were identified through informal dialogue with regional players and surveyors as well as through comments generated from the pilot test. In the pilot phase, we revised the questionnaire continually.

The final questionnaire focussed on seven types of support, which were divided into three categories. The first category, ‘regional meetings,’ included information meetings (arranged by the region), where the participants were informed about procedures, guidelines or plans for their own clinic; it also included workshops, where the participants were not only informed but also worked actively with these. Moreover, this category included visits to the clinic from the regional staff with the main purpose of providing support for the accreditation. The second category, ‘peer discussions,’ included discussions with colleagues in formal networks (e.g. GP societies, continuing training or educational upgrading) and informal discussions with GPs or staff from other clinics. The last category, ‘documents,’ consisted of accreditation documents produced by other general practice clinics for their accreditation and accreditation documents produced by others (e.g. the region or the Danish Quality Unit of General Practice).

The respondents were asked whether they had used each of the seven types of support (yes/no). Furthermore, the respondents were asked to assess the perceived usefulness of each type of support on a five-point Likert scale comprising ‘to a very large extent, to a large extent, to some extent or to a lesser extent and not at all.’ Moreover, the questionnaire included questions about general practice characteristics, including practice type and number of GP partners/staff (because a complex organisation might have different needs than a simpler one) and GP trainees (yes/no; because clinics with trainees might be more used to implement new initiatives). In the analyses, practice type was dichotomised into ‘group practice’ (defined as clinics with more than one GP owner and/or formalised cooperation with other clinics, corresponding to the Danish samarbejdspraksis or delepraksis) and ‘single-handed practice.’ This distinction was made because the need for implementation support was expected to be different in large, complex organisations than in small clinics.

### Statistical analysis

Descriptive statistics was performed to investigate the use and perceived usefulness of different types of implementation support. Usefulness was dichotomised into ‘useful (to a very large extent, to a large extent and to some extent) and not useful (to a lesser extent and not at all).’ Logistic regression analysis was used to estimate the association between use and usefulness, respectively, and the following clinic characteristics (explanatory variables): number of GP partners in the clinic (continuous variable), practice type (single-handed or group), number of staff members in the clinic (continuous variable) and GP trainees in the clinic (yes/no). The clinic characteristics were mutually adjusted in the regression model to avoid potential confounding.

The data analysis was performed using Stata statistical software IC15 (StataCorp, College Station, TX), and the level of significance was set at *p* < 0.05.

In the presentation of data, all cases were included. In the analyses, only respondents who had answered the specific outcome question were included.

## Results

**Figure 1.**
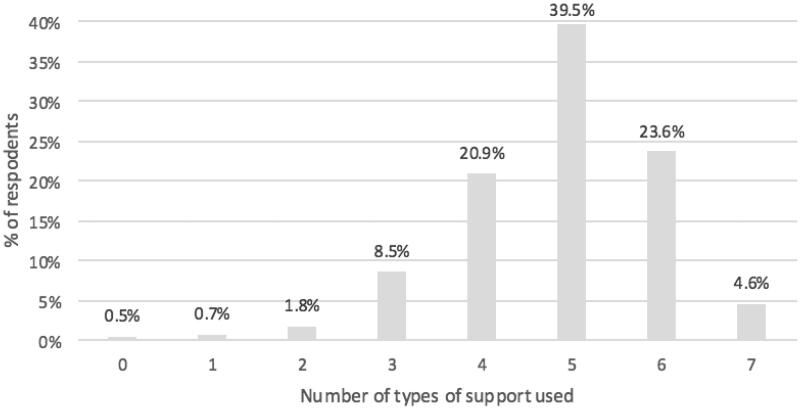
Number of different types of support used (*n* = 436). Nine respondents had missing information.

A total of 447 clinics completed and returned the questionnaire (response rate: 74%). Table 1 shows the distribution of different clinic characteristics among the respondents. Ninety-seven percent of the clinics used at least three different types of support and less than 1% did not use any type of support (Figure 1). The mean was 4.84 (confidence interval (CI): 4.74–4.95). Only few values in the dataset were missing for use and usefulness. Among the seven types of support, 11 values were missing for use, and seven values were missing for usefulness.

**Table 1.** Respondent characteristics.

	*n*	%
*Practice type*		
** **Group	323	72.3
Single-handed	124	27.7
Missing	0	0.0
*Number of GP partners in the clinic*		
1	181	40.5
2	112	25.1
3–4	116	26.0
5+	34	7.6
Missing	4	0.9
*Number of staff in the clinic*		
<4	138	30.9
4–7	193	43.2
>7	116	26.0
Missing	0	0.0
*GP trainees in the clinic*		
No	147	32.9
Yes	293	65.5
Missing	7	1.6

**Figure 2.**
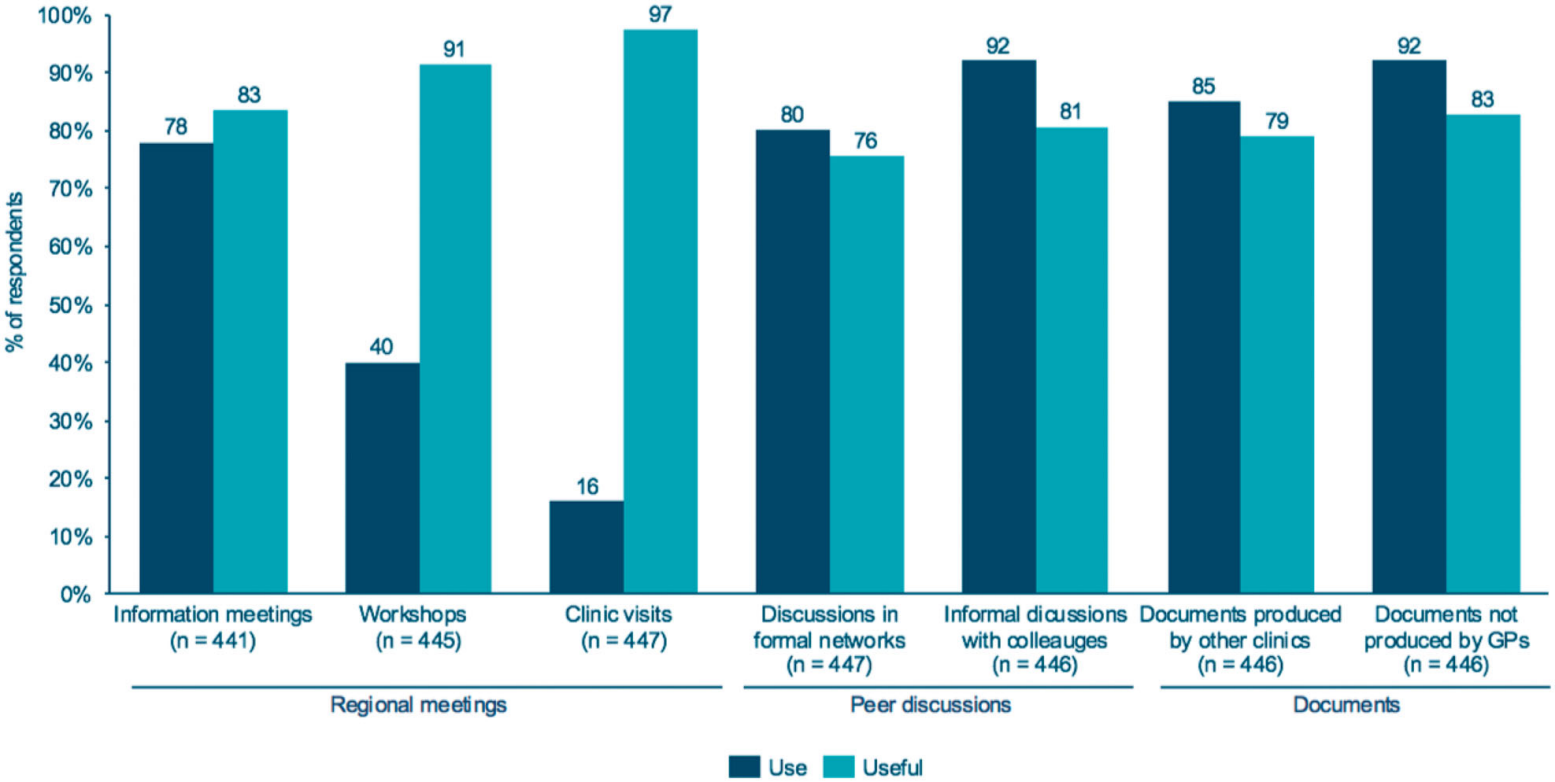
Frequency of use and perceived usefulness of implementation support.

The most frequently used types of implementation support were peer discussions and accreditation documents (Figure 2). In total, 92% of the clinics reported to have had informal discussions with colleagues from other clinics, and 80% had discussions with colleagues in formal networks. The use of accreditation documents produced by other clinics were reported by 85% of the clinics, whereas 92% had used other accreditation documents. The use of regional support varied considerably. Information meetings were attended by 78% of the clinics, 40% attended a workshop and only 16% requested a clinic visit.

Most clinics considered workshops and clinic visits to be more useful (91 and 97%, respectively) than information meetings (83%). All types of implementation support were found useful by at least 76% of the clinics (Figure 2).

Clinic characteristics were, in most cases, not significantly associated with use of implementation support (Table 2). However, compared to group practices, single-handed practices were significantly less likely to have engaged in discussions with colleagues, both in informal (odds ratio (OR) = 0.47 (CI: 0.25–0.89)) and formal networks (OR = 0.26 (CI: 0.10–0.72)). Moreover, clinics with more GP partners reported significantly more often to have engaged in informal discussions (OR = 1.95 (CI: 1.11–3.41)). In contrast, clinics with more staff members reported significantly less often to have engaged in informal discussions (OR = 0.82 (CI: 0.68–0.99)).

**Table 2.** Adjusted odds ratio (OR) for use of implementation support by clinic characteristics (95% confidence interval (CI))^a^.

	Regional meetings			Peer discussions		Documents	
	Information meetings	Workshops	Clinic visits	Discussions in formal networks	Discussions in informal networks (with colleagues)	Documents produced by other clinics	Documents produced by others
*n*	436	437	439	439	438	438	438
Practice type							
** **Group	1	1	1	1	1	1	1
** **Single-handed	0.90 (0.50; 1.64)	1.39 (0.83; 2.32)	1.27 (0.64; 2.51)	**0.47** (0.25; 0.89)	**0.26** (0.10; 0.72)	0.76 (0.38; 1.52)	0.71 (0.28; 1.81)
Number of GP partners in the clinic^b^							
** **continuous variable	1.04 (0.75; 1.43)	0.94 (0.73; 1.22)	1.03 (0.73; 1.45)	0.90 (0.65; 1.24)	**1.95** (1.11; 3.41)	0.86 (0.60; 1.25)	0.82 (0.50; 1.34)
Number of staff members in the clinic ^b^							
** **continuous variable	0.99 (0.88; 1.13)	1.03 (0.93; 1.14)	1.01 (0.88; 1.16)	0.99 (0.87; 1.13)	**0.82** (0.68; 0.99)	1.04 (0.89; 1.21)	1.10 (0.90; 1.35)
GP trainees in the clinic							
** **No	1	1	1	1	1	1	1
** **Yes	1.54 (0.90; 2.63)	1.08 (0.68; 1.72)	1.04 (0.56; 1.93)	0.93 (0.52; 1.65)	0.93 (0.41; 2.11)	1.56 (0.84; 2.91)	0.83 (0.35; 1.94)

Bold indicates a *p* value of <0.05.

a Outcome variable: Use of implementation support vs non-use of implementation support; explanatory variables = clinic charachteristics.

b The unit in these analyses is per partner/staff member.

No significant associations were found between clinic characteristics and the perceived usefulness of implementation support (data not shown).

## Discussion

### Principal findings

The clinics used a broad range of implementation support during their accreditation process, and they combined formal and informal types of support. Discussions with peers and use of accreditation documents were the most frequently used types. Support tailored to the individual clinic, including clinic visits and regional workshops, was perceived as most useful by most clinics, but these types of support were used less frequently. In most cases, clinic characteristics were neither significantly associated with the use of support nor with the perceived usefulness of the available support.

### Strengths and limitations

To our knowledge, this is the first quantitative study to investigate implementation support during accreditation in the general practice setting. Strengths include hand distribution of the questionnaire to all clinics immediately after their survey visit and the high response rate compared to several other questionnaire studies in this setting [16,17]. In addition, only few respondents did not answer the questions which were used for analyses in this study. Furthermore, the study combined the estimated use of implementation support with an evaluation of the quality as perceived by the GPs. This combination provides good possibilities for using the findings of this study to inform the design of future implementation support.

One limitation is the embedded risk of recall bias as both use and usefulness of support were measured subjectively. The clinics are recommended to begin the preparation for the accreditation survey at least six months before their accreditation survey [6]. This entails a risk that the respondents may not remember correctly which type of support they had used and how useful they found it. The study attempts to address the risk of recall bias by asking the GP most involved in the accreditation process to complete the questionnaire. However, this induces a risk of selection bias, because the most deeply involved GP might be more positive towards the accreditation process in the clinic. Therefore, both the risk of recall bias and selection bias should be borne in mind when interpreting the results; especially, it might be of significance that the usefulness was assessed after completion of the accreditation process of the clinics and not immediately after receipt of the intervention support.

Accreditation comprises fulfilment of a broad range of quality standards, making the accreditation process very complex. The multiple aims tend to result in challenges with measuring the effect of accreditation [18,19]. Likewise, it is difficult to assess how the different types of support assist the clinics in their accreditation process, and whether use of support of self-perceived usefulness is associated with a positive accreditation status after the survey. Our study does not provide information about what the clinics might gain from using different types of implementation support.

### Findings in relation to other studies

A previous study has confirmed that general practice clinics use different types of support to improve their understanding of the standards and that the availability of useful implementation support is important [6]. The same study also found that the value of regional information meetings depended on how well the information matched the specific context in the clinic, e.g. whether the timing of the meeting suited their own process of working with the standards [6]. This could be a reason why tailored support, in our study, was considered more useful than more general types of support.

Interventions with high degree of user engagement are often more likely to induce changes. In our study, the activities requiring more time and engagement were also considered more useful. However, it still needs be recognised that different clinics have different priorities, and not all clinics can be expected to choose the more demanding implementation support activites.

Our study found no significant associations between clinic characteristics and the experienced usefulness of the support provided. This suggests that the right timing of the implementation support is far more important than clinic characteristics for benefitting from the support. The importance of timing could also explain why we, in our study, found no significant associations between clinic characteristics and perceived usefulness of support. This could also explain why the use of accreditation documents and peer discussions were found more useful as these types of support are more flexible in terms of timing.

Still, our study found some variations among the included clinics. The association between different clinic characteristics and information-seeking behaviour has been investigated in previous research [16,20]. One study found that GPs working in a single-handed practice tended to seek advice from peers less often than GPs working in a group practice, although they did not use other types of information sources [16]. This supports the finding from our study that discussions with colleagues outside the clinic (both in informal and formal networks) were more frequent in group practices compared to single-handed practices. The higher use of peer discussions in group practices may be due to higher complexity in this type of clinic and thus higher demands of dialogue and more established traditions of discussing with peers. However, it may also just reflect that clinics with more GPs have a greater chance that one of them will discuss accreditation with a GP from another clinic than clinics with only one GP.

It is likely that the use of tailored types of implementation support could be promoted by ensuring that the clinics are aware of these options [6]. Different strategies were used, with varying degrees of proactivity, when the regions informed the clinics about the possibility of receiving a visit [15].

The impact of peers for making changes in general practice is also well known in the literature; one example is the positive impact on practice facilitation [7,8]. The importance of peers is also acknowledged in the accreditation survey as one of the surveyors is a GP [6]. The idea of using peers is not limited to GPs; it is widely used in the healthcare system [21]. The underlying mechanism in peer theory is that the message will be understood better if it comes from someone who is like yourself [22]. This is due to more precise communication and high degree of trust. It can be argued that the importance of being a peer is particularly relevant among GPs since general medicine is a profession with a high degree of expertise, and because knowledge about the management of GP clinics is necessary to understand the barriers and facilitators for GPs’ professional behaviour [22].

General practice accreditation was terminated in Denmark by 1 January 2019, and each clinic completed only one accreditation survey each. Therefore, the results of this study relate to the first and so far last round of accreditation. The need for external support to implement accreditation standards will presumably diminish in later rounds, provided that the requirements for accreditation do not change substantially. The results of this study therefore relate to implementation of new initiatives in general, rather than to implementation of repeated accreditation rounds.

### Implications

Our study suggests that GPs find it meaningful and especially useful to engage in dialogue with peers outside their clinic during a process of change. It should be investigated how peer discussions could be facilitated in future quality interventions.

Our study shows that the clinics need different types of support, and the combination of support types depends on their needs and willingness to invest resources in the support. Therefore, a multifactorial approach to implementation support should be endeavoured in future quality interventions.

Based on the findings in this study, it is recommended that future quality improvement initiatives should provide support that is ‘multifaceted’ (to target the various needs of the clinics), is ‘independent of timing’ (so that each clinic can use it whenever suitable) and includes support based on ‘collaboration between clinics’ (as peers increase the perceived usefulness of the provided support).
